# Prevalence of Seizures in Children Diagnosed With Neurodevelopmental Disorders

**DOI:** 10.7759/cureus.63765

**Published:** 2024-07-03

**Authors:** Osama Y Muthaffar, Abrar Y Abbar, Mohammed T Fitaih

**Affiliations:** 1 Department of Pediatrics, Faculty of Medicine, King Abdulaziz University, Jeddah, SAU; 2 Department of Pediatrics, East Jeddah General Hospital, Ministry of Health, Jeddah, SAU; 3 General Practice, King Abdulaziz International Airport Medical Control Center, Ministry of Health, Jeddah, SAU

**Keywords:** attention deficit hyperactivity disorder (adhd), saudi arabia, epilepsy, seizures, autism spectrum disorder, intellectual disability, global developmental delay, cerebral palsy, neurodevelopmental disorders

## Abstract

Introduction

Neurodevelopmental disorders (NDDs) typically emerge in early childhood and have a profound impact on the development of the nervous system, leading to various neurological challenges in cognition, communication, social interaction, motor skills, and behavior. These disorders arise from disruptions in brain development mechanisms. NDDs include conditions such as cerebral palsy (CP), global developmental delay (GDD), intellectual disability (ID), attention-deficit/hyperactivity disorder (ADHD), and autism spectrum disorder (ASD), with ADHD and ASD being the most prevalent. However, there is a lack of comprehensive research on the causes of NDDs in children receiving care at tertiary hospitals in Saudi Arabia. Therefore, in this study, we aim to investigate the characteristics of patients with NDDs and explore the association between NDDs and seizures. It also focuses on identifying specific risk factors that may influence the relationship between NDDs and seizures.

Methods

We conducted a retrospective cross-sectional study at the pediatric neurology and developmental assessment clinic of King Abdulaziz University Hospital in Jeddah, Saudi Arabia. The study involved a review of electronic medical records from January 2021 to May 2023 for 200 pediatric patients who attended the clinic for NDD and seizures. Descriptive statistics summarized the data, using frequencies and percentages for categorical variables, and mean ± standard deviation for quantitative variables. The chi-square test identified differences between qualitative variables, with a significance threshold of p < 0.05.

Results

The study sample comprised 200 children ranging in age from one month to 14 years, with the majority of patients being from Jeddah city. Participants were categorized into four age groups: 17.0% (n=34) were aged between one month and three years, 18.5% (n=37) were aged between three and six years, 55.0% (n=110) were aged between six and 12 years old, and 9.5% (n=19) were aged between 12 and 14 years. The NDD subtypes identified were ASD 9.5%, ADHD 16.0%, CP 8.5%, GDD 30.5%, ID 5.5%, and 30% had multiple types of NDD. Generalized tonic-clonic seizures were the most common type observed.

Conclusion

Children with NDDs exhibit a high prevalence of seizures, with the age of the patient and consanguinity emerging as significant influencing factors in this correlation. Among the key findings is an emphasis on the importance of early detection and intervention for children with NDDs at higher risk of developing seizures. Overall, the study sheds light on the characteristics of NDD patients and their association with seizures, contributing to a better understanding of the complex relationship between NDDs and seizure occurrence. It also emphasizes the need for comprehensive assessment and management strategies that consider seizures in children with NDDs.

## Introduction

Neurodevelopmental disorders (NDDs) encompass a group of conditions that typically emerge in early childhood and impact the development of the nervous system. These disorders affect various aspects of neurological functioning, including cognition, communication, social interaction, motor skills, and behavior [1]. They arise due to disruptions in the mechanisms governing brain development. NDDs include conditions such as cerebral palsy (CP), global developmental delay (GDD), intellectual disability (ID), attention-deficit/hyperactivity disorder (ADHD), and autism spectrum disorder (ASD). ASD and ADHD are among the most prevalent NDDs [1].

ADHD is the most common neurobehavioral diagnosis in children worldwide, diagnosed based on the Diagnostic and Statistical Manual of Mental Disorders, Fourth Edition (DSM-IV) criteria by psychologists, parents, and teachers. ASD is one of the most common NDDs globally, characterized by persistent deficits in social communication and interaction, language development, and restricted repetitive patterns of behaviors and interests [2]. The exact cause of ASD remains unknown. ID refers to limitations in a child's ability to learn and function at an expected level in daily life. CP is a frequent cause of motor disability in children, defined as a group of permanent non-progressive movement and posture disorders resulting from brain abnormalities during fetal or infant development [2]. GDD is a general term used to describe significant delays in two or more developmental domains (daily living activities, motor skills, speech/language, cognitive abilities, and social/emotional skills) in children under five years old. The exact prevalence of GDD is unknown but estimated to be 1-3% [3].

Seizures are a common condition affecting 6.5 per 1000 population and are characterized by abnormally high levels of synchronous neuronal activity in the brain, accompanied by temporary indications or symptoms [4]. Seizures can be classified as focal, generalized, or of unknown onset. Generalized seizures involve both brain hemispheres from the beginning, while focal seizures start in a specific area or hemisphere. Generalized seizures can further be classified as motor or non-motor (absence) onset. Focal seizures can be related to impaired awareness or exhibit motor (tonic, clonic, atonic, or myoclonic activity) or non-motor (behavioral arrest, cognitive, emotional, sensory, or autonomic characteristics) symptoms. It is important to inquire about premonitory symptoms, which may act as an aura, in children with generalized tonic-clonic seizures, as focal seizures can progress to bilateral convulsive activity [5-7].

Limited research has been conducted on the causes of NDDs in children attending tertiary care hospitals. Similarly, there is a lack of studies exploring the prevalence of seizures among children with NDDs. A recent study conducted at King Fahad Specialist Hospital in Dammam, Saudi Arabia, examined the prevalence of seizures in pediatric patients with GDD and found that 56% of Saudi children with GDD had epilepsy. The study also identified low birth weight (22%) and consanguineous marriage (57%) as factors associated with NDD. The causes of NDD included abnormal neuroradiology in 58% of cases, abnormal electroencephalogram (EEG) in 45%, genetic problems in 40%, and metabolic issues in 25% [1].

The present study aims to investigate the prevalence of seizures in children with NDDs who visited a pediatric neurology and development clinic, as well as examine the demographic characteristics of these patients.

## Materials and methods

Ethical considerations

The study was approved by the Research Ethics Committee at the Faculty of Medicine, King Abdulaziz University (reference number: 108-24). Participants' confidentiality was strictly maintained. All participants, through their parents, received a detailed explanation of the study's purpose, procedures, and potential benefits and risks. Informed consent was obtained from the parents or legal guardians before participation. The study adhered to the principles outlined in the Declaration of Helsinki and ensured that data were anonymized to protect the identities of the participants.

Study design

This retrospective cross-sectional study was conducted at the pediatric neurology clinic at King Abdulaziz University Hospital in Jeddah, Saudi Arabia. The study included a sample of 200 pediatric patients, aged one month to 14 years. The sample size was calculated based on the number of patients visiting the pediatric neurology clinic using Raosoft Sample Size Calculator software (Raosoft Inc., Seattle, Washington, United States). It also included patients visiting the clinic between January 2021 and May 2023. The study design was constructed to obtain the prevalence of seizures among children with NDDs within the specified period, allowing for the analysis of various demographic and clinical factors including the patient's sex, age, seizure semiology, clinical diagnoses, developmental assessment, and electrographic and neuroradiological data.

Participant selection

Participants were selected using convenience sampling. All children referred to the pediatric neurology clinic and who underwent a multidisciplinary developmental assessment were considered for inclusion. The developmental assessment included a comprehensive history taking and a developmental pediatric examination. The inclusion criteria were: (i) Children of both genders, (ii) aged one month to 14 years, (iii) children diagnosed with ASD, ADHD, CP, GDD, and ID, which was (iv) confirmed by a pediatric doctor using magnetic resonance imaging (MRI), EEG, or genetic testing. Patients were excluded if they had incomplete information in their medical records or did not complete the full developmental assessment.

Data collection 

Data were collected from the hospital's electronic medical records system. Variables included demographic characteristics (age and gender), clinical diagnosis (ASD, ADHD, CP, GDD, ID), type of seizures, and MRI and EEG findings. Additional details such as developmental milestones were also recorded.

Statistical analysis

Data were meticulously entered into a preformatted Microsoft Excel spreadsheet (version 2010; Microsoft Corporation, Redmond, Washington, United States) and updated concurrently to ensure accuracy. The spreadsheet was constructed based on the study's objectives. The data were subsequently imported into IBM SPSS Statistics for Windows, Version 22.0 (Released 2013; IBM Corp., Armonk, New York, United States) for statistical analysis. Descriptive statistics were used to summarize the data, with frequencies and percentages computed for categorical variables, and mean ± standard deviation (SD) (minimum-maximum) for quantitative variables. The chi-square test was employed to examine differences between qualitative variables, with a p-value of < 0.05 considered statistically significant. The analysis aimed to identify significant associations between the prevalence of seizures and factors such as age, gender, type of NDD, MRI, and EEG findings.

## Results

A total of 200 pediatric patients diagnosed with NDD were included in the study. Participants were divided into four categories based on age: 17.0% (n=34) patients were in the age group of one month to three years of age, 18.5% (n=37) were aged between three and six years, 55.0% (n=110) of patients were aged between six and 12 years, and 9.5% (n=19) were aged between 12 and 14 years (Table 1).

**Table 1:** Distribution of participants according to age (N=200)

Age	Frequency	Percentage	p-value
1 month-3 years	34	17.0%	0.001
>3-6 years	37	18.5%	
>6-12 years	110	55.0%	
>12-14 years	19	9.5%	

Regarding NDD subtypes, ASD was reported in 9.5% (n=19) of the participants, ADHD was reported in 16.0% (n=32), CP was reported in 8.5% (n=17), GDD was reported in 30.5% (n=61), ID was reported in 5.5% (n=11), and more than one type of NDD was reported in 30% (n=60) (Table 2).

**Table 2:** Types of neurodevelopmental disorders in the participants (N=200)

Neurodevelopmental disorder	Frequency	Percentage
Autism spectrum disorder	19	9.5%
Attention-deficit/hyperactivity disorder	32	16.0%
Cerebral palsy	17	8.5%
Global developmental delay	61	30.5%
Intellectual disability	11	5.5%
More than one	60	30.0%

The relation between NDDs and seizures was reported in 31.6% (n=6/19) of ASD patients, 62.5% (n=20/32) of ADHD patients, 52.9% (n=9/17) of CP patients, 49.2% (n=30/61) of GDD patients, 36.4% (n=4/11) of ID patients, and 58.3% (n=35/60) of patients with more than one type of NDD (Table 3).

**Table 3:** The relation between neurodevelopmental disorders and seizures ASD: autism spectrum disorder; ADHD: attention-deficit/hyperactivity disorder; CP: cerebral palsy; GDD: global developmental delay; ID: intellectual disabilities

Neurodevelopmental disorders	Had seizures (52.0%; n=104)		Did not have seizures (48.0%; n=96)	
	Frequency	Percentage	Frequency	Percentage
ASD (n=19)	6/19	31.6%	13/19	68.4%
ADHD (n=32)	20/32	62.5%	12/32	37.5%
CP (n=17)	9/17	52.9%	8/17	47.1%
GDD (n=61)	30/61	49.2%	31/61	50.8%
ID (n=11)	4/11	36.4%	7/11	63.6%
More than one (n=60)	35/60	58.3%	25/60	41.7%

Of the 19 participants in the ASD group, 89.5% (n=17) had seizures with 29.4% (n=5/19) of these having focal seizure type impaired motor awareness and 70.6% (n=12/17) having generalized seizures; of the generalized seizures, 75% (n=9/12) were GTC and 25% (n=3/12) were absence. The prevalence of seizures in children with ASD is presented in Table 4.

**Table 4:** Prevalence of seizure in children with ASD ASD: autism spectrum disorder; GTC: generalized tonic-clonic

Parameter	Frequency	Percentage
Children with ASD	19/200	9.5%
Seizures in children with ASD	17/19	89.5%
Focal seizure	5/17	29.4%
Impaired awareness motor	5/5	100%
Generalized seizure	12/17	70.6%
GTC	9/12	75.0%
Absence	3/12	25.0%

Of the 32 participants in the ADHD group, 84.4% (n=27) had seizures, of which 29.6% (n=8/27) had focal seizures and 70.4% (n=19/27) had generalized seizures. Focal seizures included impaired awareness motor (50.0%, n=4/8), impaired awareness non-motor (25.0%, n=2/8), and focal bilateral tonic (25.5%, n=2/8). The generalized seizure included GTC (78.9%, n=15/19), myoclonic (10.5%, n=2/19), and absence (10.5%, n=2/19). The prevalence of seizures in children with ADHD is presented in Table 5.

**Table 5:** Prevalence of seizure in children with ADHD ADHD: attention-deficit/hyperactivity disorder; GTC: generalized tonic-clonic

Parameter	Frequency	Percentage
Children with ADHD	32/200	16.0%
Seizures in children with ADHD	27/32	84.4%
Focal seizure	8/27	29.6%
Impaired awareness motor	4/8	50.0%
Impaired awareness non-motor	2/8	25.0%
Focal bilateral tonic	2/8	25.0%
Generalized seizure	19/27	70.4%
GTC	15/19	78.9%
Myoclonic	2/19	10.5%
Absence	2/19	10.5%

Of the participants in the GDD group, 27.9% (n=17/61) had focal seizures and 69.6% (n=39/56) had generalized seizures. The focal seizures included 70.6% (n=12/17) impaired awareness motor and 29.4% (n=5/17) focal bilateral tonic. The generalized seizure included GTC (51.3%, n=20/39), myoclonic (25.6% n=10/39), GT (15.4%, n=6/39), and absence (7.7%, n=3/39). Also, 91.8% (n=56/61) of patients with GDD had other forms of seizures. The prevalence of seizures in children with GDD is presented in Table 6.

**Table 6:** Prevalence of seizure in children with global developmental delay GDD: global developmental delay; GTC: generalized tonic-clonic; GT: generalized tonic

Parameter	Frequency	Percentage
GDD	61/200	30.5%
Focal onset (n=17)		
Impaired awareness motor	12/17	70.6%
Focal bilateral tonic	5/17	29.4%
Generalized onset (n=39)		
GTC	20/39	51.3%
Myoclonic	10/39	25.6%
GT	6/39	15.4%
Absence	3/39	7.7%
Other (Unknown/unclassified/mixed or other forms of seizure)	56/61	91.8%

Of the 11 participants in the ID group, 100% (n=11) had seizures of which 36.4% (n=4/11) had focal seizures and 63.6% (n=7/11) had generalized seizures. The focal seizures included impaired motor awareness (50.0%, n=2/4), impaired awareness non-motor (25.0%, n=1/4), and focal bilateral tonic (25.0%, n=1/4). Generalized seizures included GTC (85.7%, n=6/7) and absence (14.3%, n=1/7). The prevalence of seizure in children with ID is presented in Table 7.

**Table 7:** Prevalence of seizure in children with intellectual disabilities ID: intellectual disabilities; GTC: generalized tonic-clonic

Parameter	Frequency	Percentage
Children with ID	11/200	5.5%
Seizures in children with ID	11/11	100%
Focal seizure	4/11	36.4%
Impaired awareness motor	2/4	50.0%
Impaired awareness non-motor	1/4	25.0%
Focal bilateral tonic	1/4	25.0%
Generalized seizure	7/11	63.6%
GTC	6/7	85.7%
Absence	1/7	14.3%

Of the 17 participants in the CP group, 70.6% (n=12) had seizures of which 33.3% (n=4/12) of participants had focal seizures and 66.7% (n=8/12) had generalized seizures. The focal seizure included 100% (n=4/4) with impaired motor awareness. The generalized seizure included GTC (62.5%, n=5/8) and GT (37.5%, n=3/8). The prevalence of seizures in children with CP is presented in Table 8.

**Table 8:** Prevalence of seizure in children with cerebral palsy CP: cerebral palsy; GTC: generalized tonic-clonic; GT: generalized tonic

Parameter	Frequency	Percentage
Children with CP	17/200	8.5%
Seizures in children with CP	12/17	70.6%
Focal seizure	4/12	33.3%
Impaired awareness motor	4/4	100%
Generalized seizure	8/12	66.7%
GTC	5/8	62.5%
GT	3/8	37.5%

Of the 60 participants with more than one type of developmental disorder, 95.0% (n=57) had seizures, of which 29.8% (n=17/57) had focal seizures, 63.2% (n=36/57) had generalized seizures, and 7.0% (n=4/57) had both generalized and focal seizures (Table 9).

**Table 9:** Prevalence of seizure in children with more than one type of developmental disorder

Parameter	Frequency	Percentage
Children with more than one type of developmental disorder	60/200	30.0%
Seizure in children with more than one type of developmental disorder	57/60	95.0%
Focal seizure	17/57	29.8%
Generalized seizure	36/57	63.2%
Both generalized and focal seizure	4/57	7.0%

Diagnostic tests done on NDD reported 55.0% abnormalities in MRI and 73.0% abnormalities in EEG investigations (Table 10).

**Table 10:** Diagnostic tests utilized

Investigation	Normal, n (%)	Abnormal, n (%)
MRI	90 (45.0%)	110 (55.0%)
EEG	54 (27.0%)	146 (73.0%)

## Discussion

The present study investigated the prevalence of seizures in children with NDDs, which is currently an understudied topic. This was a retrospective hospital-based study conducted at a tertiary care hospital in Jeddah, Saudi Arabia. The study included 200 participants, with 17.0% aged between one month and three years, 18.5% aged between three and six years, 55.0% aged between six and 12 years, and 9.5% aged between 12 and 14 years. A study by Berg et al. found that cognitive impairment was more prevalent in participants who experienced seizures before the age of five [8].

In our study, the prevalence of NDD subtypes was as follows: 9.5% had ASD (n=19), 16.0% had ADHD (n=32), 8.5% had CP (n=17), 30.5% had GDD (n=61), 5.5% had ID (n=11), and 30% had more than one type of NDD (n=60). A previous study in Sweden reported an increased risk of developmental coordination disorder, ASD, and ID in children with a history of seizures around the ages of nine or 12 years [9]. Another study found that one-third of children with a history of seizures at the age of four to five years had neurodevelopmental impairments or developmental issues related to attention, hyperactivity, and behavior [10]. Generalized seizures were the most common seizure type in our study group, followed by focal impaired awareness motor seizures, which is consistent with previous studies [11-13].

Regarding the relationship between NDD and seizures, we found that 89.5% of children with ASD had seizures, 84.4% of children with ADHD had seizures, 70.6% of children with CP had seizures, and 100% children with ID had seizures. Our results also revealed a high prevalence of consanguineous marriages among the participants, consistent with a previous prospective study that found consanguinity in 29.7% of participants with recurrent seizures [14]. Another study reported that 61.6% of children with NDD had parents who were related [15].

In the ADHD group, 84.4% of participants (n=27/32) had seizures. Among those with seizures, 29.6% (n=8/27) had focal seizures and 70.4% (n=19/27) had generalized seizures. Focal seizures included impaired awareness motor (50.0%, n=4/8), impaired awareness non-motor (25.0%, n=2/8), and focal bilateral tonic (25.0%, n=2/8). Generalized seizures included GTC (78.9%, n=15/19), myoclonic (10.5%, n=2/19), and absence seizures (10.5%, n=2/19). Previous studies have suggested a potential genetic factor shared between seizures and ADHD [16]. Studies conducted in Denmark and Taiwan have reported an association between seizures and a higher risk of ADHD [16,17].

It is important to note that our study has limitations. It is a retrospective cross-sectional study with a convenience sample collected from children under 15 years of age who were followed in the pediatric neurology department of the hospital. The retrospective nature of the data and the relatively small sample size may limit the generalizability of our results to the entire population of Saudi Arabia. Additionally, selection bias may have influenced our findings.

## Conclusions

This study highlights the prevalence of seizures in children with NDDs including ADHD, ASD, CP, ID, and GDD. NDDs are a group of disorders characterized by impairments in cognition, communication, behavior, and motor skills due to abnormal brain development. By examining the prevalence of seizures in these patients and considering factors such as age, seizure type, MRI and EEG findings, and consanguineous marriage, several key insights have emerged. Based on our cohort, we found a high prevalence of seizures among children with NDDs, underscoring the need to recognize and address seizures within the context of NDDs, which has implications for their management and treatment. Age was identified as a significant factor influencing seizure prevalence in NDD patients. This finding emphasizes the importance of early identification and intervention for children with NDDs at higher risk of developing seizures such as younger age of presentation, abnormal EEG, and positive consanguinity.

Additionally, consanguineous marriage was identified as a significant factor in the prevalence of seizures among children with NDDs. The high prevalence of consanguineous marriages among study participants suggests a potential genetic component contributing to the increased seizure risk in this population. This finding highlights the importance of genetic counseling and awareness of familial risk factors in assessing and managing children with NDDs. Overall, this study enhances our understanding of the relationship between seizures and NDDs, providing valuable insights into prevalence and associated factors. The findings emphasize the need for comprehensive assessment and management strategies that consider seizures in children with NDDs, taking into account their age, genetic factors, and other relevant clinical characteristics. Such knowledge can inform healthcare professionals and guide the development of targeted interventions to improve outcomes and quality of life for children with NDDs affected by seizures.
